# Delocalization of exciton and electron wavefunction in non-fullerene acceptor molecules enables efficient organic solar cells

**DOI:** 10.1038/s41467-020-17867-1

**Published:** 2020-08-07

**Authors:** Guichuan Zhang, Xian-Kai Chen, Jingyang Xiao, Philip C. Y. Chow, Minrun Ren, Grit Kupgan, Xuechen Jiao, Christopher C. S. Chan, Xiaoyan Du, Ruoxi Xia, Ziming Chen, Jun Yuan, Yunqiang Zhang, Shoufeng Zhang, Yidan Liu, Yingping Zou, He Yan, Kam Sing Wong, Veaceslav Coropceanu, Ning Li, Christoph J. Brabec, Jean-Luc Bredas, Hin-Lap Yip, Yong Cao

**Affiliations:** 1 State Key Laboratory of Luminescent Materials and Devices, Institute of Polymer Optoelectronic Materials and Devices, School of Materials Science and Engineering, South China University of Technology, 381 Wushan Road, 510640 Guangzhou, P. R. China; 2 Innovation Center of Printed Photovoltaics, South China Institute of Collaborative Innovation, 523808 Dongguan, P.R. China; 3 School of Chemistry and Biochemistry and Center for Organic Photonics and Electronics, Georgia Institute of Technology, 30332-0400 Atlanta, GA USA; 4 Department of Chemistry and Biochemistry, The University of Arizona, Tucson, AZ 85721-0088 USA; 5 Department of Mechanical Engineering, The University of Hong Kong, Pokfulam, Hong Kong P. R. China; 6 National Synchrotron Radiation Laboratory, University of Science and Technology of China, 230029 Hefei, P. R. China; 7 Department of Materials Science and Engineering, Monash University, Clayton, VIC 3800 Australia; 8 Australian Synchrotron, ANSTO, Clayton, VIC 3168 Australia; 9 Department of Chemistry and Physics, Hong Kong University of Science and Technology (HKUST), Clear Water Bay, Kowloon, Hong Kong P. R. China; 10 Institute of Materials for Electronics and Energy Technology (i-MEET), Department of Materials Science and Engineering, Friedrich-Alexander-Universität Erlangen-Nürnberg, Martensstr. 7, 91058 Erlangen, Germany; 11 Helmholtz‐Institute Erlangen‐Nürnberg (HI ERN), Immerwahrstrasse 2, 91058 Erlangen, Germany; 12 College of Chemistry and Chemical Engineering, Central South University, 410083 Changsha, P. R. China; 13 National Engineering Research Center for Advanced Polymer Processing Technology, Zhengzhou University, 450002 Zhengzhou, P. R. China

**Keywords:** Solar cells, Atomic and molecular interactions with photons

## Abstract

A major challenge for organic solar cell (OSC) research is how to minimize the tradeoff between voltage loss and charge generation. In early 2019, we reported a non-fullerene acceptor (named Y6) that can simultaneously achieve high external quantum efficiency and low voltage loss for OSC. Here, we use a combination of experimental and theoretical modeling to reveal the structure-property-performance relationships of this state-of-the-art OSC system. We find that the distinctive π–π molecular packing of Y6 not only exists in molecular single crystals but also in thin films. Importantly, such molecular packing leads to (i) the formation of delocalized and emissive excitons that enable small non-radiative voltage loss, and (ii) delocalization of electron wavefunctions at donor/acceptor interfaces that significantly reduces the Coulomb attraction between interfacial electron-hole pairs. These properties are critical in enabling highly efficient charge generation in OSC systems with negligible donor-acceptor energy offset.

## Abstract (web summary)

Y6, as a non-fullerene acceptor for organic solar cells, has attracted intensive attention because of the low voltage loss and high charge generation efficiency. Here, Zhang et al. find that the delocalization of exciton and electron wavefunction due to strong π-π packing of Y6 is the key for the high performance.

## Introduction

Bulk-heterojunction organic solar cells (OSCs) are light-weight, flexible, non-toxic and aesthetic devices that show great potential as a new technology for portable power sources and building-integrated photovoltaic applications^1–4^. However, the OSC power conversion efficiencies (PCEs) still lag behind those of their inorganic counterparts, in part because of the excitonic nature of organic semiconductor materials. Additional energy is typically required for electron-hole separation at the donor/acceptor interfaces, which leads to large energy (voltage) loss between the optical gap *E*_opt_^onset^ and e*V*_OC_, where *V*_OC_ denotes the open-circuit voltage. This deficiency manifests particularly in OSCs that use fullerene derivatives as electron acceptors, with energy losses typically in the range of 0.8–1.0 eV depending on the choice of polymer donors. As a result, the PCEs of the best fullerene-based OSCs are limited to only ~10–11%^5–8^. Over the past few years, tremendous efforts have been dedicated to the development of fullerene replacements, such as n-type polymers and small molecules with fused-ring structures. These non-fullerene acceptors (NFAs) not only exhibit stronger light absorption and wider tunability of spectral range and energy levels, but also enable much reduced energy losses below ~0.6 eV, which lead to OSCs with PCEs outperforming those based on fullerenes^9–11^. Among these NFAs, the family of the A-D-A type molecules (where A denotes an electron-poor/acceptor moiety and D, an electron-rich/donor moiety), typically composed of linearly fused conjugated ring as the middle D core, such as indacenodithienothiophene (IDT), have shown promising results with PCEs up to 13–14%^12–14^.

**Fig. 1:**
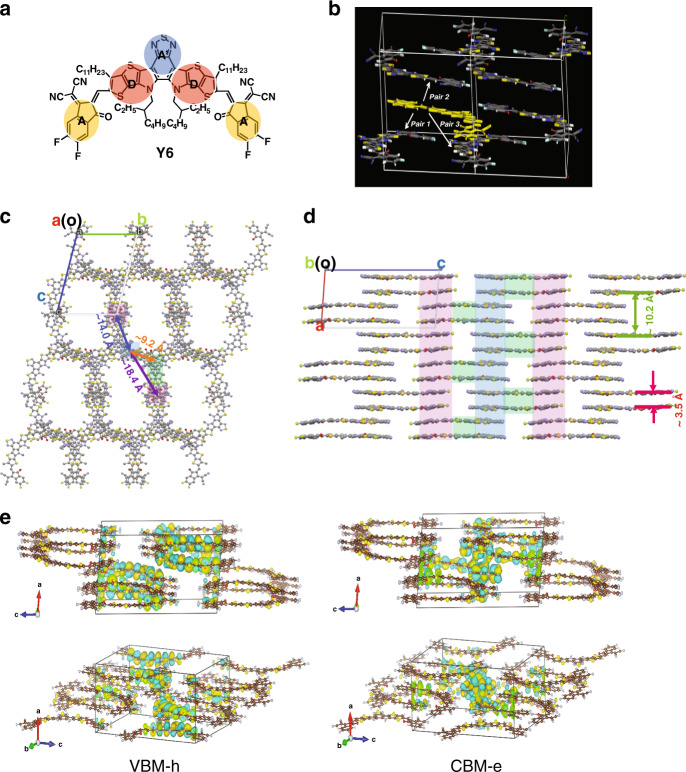
Molecular and single-crystal structures, and wavefunction distribution of Y6. **a**–**e** Molecular structure of Y6 (**a**). **b** Molecular pairs in the Y6 single crystal. **c** Top and **d** side views of the extended-crystal structure (the blue column is the stack of end groups in the ***b*** direction, the pink column is the stack of end groups in the ***c*** direction, and the green one is a molecular packing pair of D–A′ fragment). **e** Calculated valence band maximum (VBM) for hole (left) and conduction band minimum (CBM) for electron (right) wavefunctions at the Γ point (center of the Brillouin zone) in the Y6 single crystal.

The development of OSCs with NFAs based on the A-D-A molecular design appears, however, to have reached a bottleneck. Although the energy levels of NFA-based acceptors can be easily tuned to minimize the offset between the polymer donor and NFA ionization energies or electron affinities (or, in less rigorous terminology, between their highest occupied molecular orbital (HOMO) or lowest unoccupied molecular orbital (LUMO) energies) in order to reduce voltage loss^15,16^, there remains a significant tradeoff between achieving low voltage loss and high charge-generation efficiency^17,18^. In early 2019, we reported an acceptor Y6 based on a core unit, dithienothiophen[3.2-b]-pyrrolobenzothiadiazole (TPBT), corresponding to a A–DA′D–A molecular configuration with a curved molecular geometry (Fig. 1a)^19^. When blended with a donor polymer PBDB-T-2F (Supplementary Fig. 1), the device shows very high photocurrent generation quantum efficiency (EQE of ~85%, IQE of ~95%) that extends to ~920 nm. Under simulated sunlight (AM 1.5 G) the device shows a high fill factor (FF) of 73.3% and a *V*_OC_ of ~0.82 V, and the overall PCE is ~15.7%. Material and device characterization data can be found in the Supplementary Figs. 2 and 3, and Supplementary Tables 1 and 2. The HOMO offset between donor and acceptor is ~0.05 eV. We calculate a voltage loss of 0.53 V and 0.67 V by using *E*_g_^onset^ and *E*_opt_^edge^ as the optical gap, respectively, and it exhibits a low non-radiative voltage loss (∆*V*_OC,nr_) further discussed below. We note that, while a number of previous studies have reported lower ∆*V*_OC,nr_ values^16,20^, most of the systems under consideration are either based on wide- or intermediate-gap systems with high-energy charge transfer (CT) states (and we recall that ∆*V*_OC,nr_ correlates inversely with the CT-state energy^21,22^), otherwise the devices show lower charge generation efficiency (EQE/IQE; Supplementary Table 3). However, PBDB-T-2F:Y6 based OSCs show an EQE onset extending to 920 nm, and it is very rare for such a low-gap system to simultaneously achieve a low ∆*V*_OC,nr_ and an extremely high EQE (Supplementary Fig. 4), which were also found in other reports based on Y6 and Y6 derivative systems^23–25^.

This material design have triggered extensive investigations over the past year aiming to further improve the performance of OSCs by fine tuning the Y6-based acceptor molecular structures^26–28^, introducing ternary blend systems^29–31^ and using new donor polymers^32,33^, leading to further increase in the device efficiency. Although these material and device engineering strategies are effective in improving Y6-based OSCs, there were few research efforts that focused on revealing the origin of the remarkable performances enabled by this molecular design^34^. It has been proposed that studies of the single-crystal structure of Y6-based molecules could be useful in revealing the intrinsic properties of the acceptor^25,35^. However, to better evaluate the structure-property relationship of the OSCs, the molecular packing of the acceptor in the BHJ film and how such packing can affect the electronic processes at the acceptor/donor interface need to be studied in order to provide better understanding on the origin of the high-performance devices.

Therefore, in this report, we study Y6-based OSCs using a combination of single-crystal investigations, thin film morphology analysis (grazing incidence wide-angle X-ray scattering, GIWAXS), spectroscopy, as well as theoretical simulations, to uncover the structure-property-performance relationships in these materials. We find that the distinctive π-π molecular packings of the Y6 molecules not only exist in the single crystal but also the spin-coated films, which facilitates: (i) the formation of delocalized and emissive excitons that can be effectively populated from the CT state, leading to small non-radiative voltage loss; (ii) efficient delocalization of electron wavefunctions at the donor/acceptor interfaces, which significantly reduces the interfacial Coulomb attraction of the CT state. These findings help explain the highly efficient charge generation in Y6-based OSCs even though the driving force for exciton dissociation is small.

## Results

### Single-crystal structure and wavefunction distribution

Since the electronic and optical properties of organic semiconductor materials can be strongly affected by their structural properties^36^, we begin by studying the intrinsic molecular packing of Y6 using single-crystal X-ray diffraction (see “Methods” for detail). As shown in Supplementary Fig. 5, Y6 exhibits a planar configuration with intramolecular noncovalent locks coming from S^…^O=C interactions at the D–A junction^37^; the curved Y6 molecule favors intermolecular “face-on” stacking through π-π interactions among the end groups (with the molecular planes ~3.5 Å apart). As shown in Fig. 1c, d, we find that a continuous and regular three-dimensional (3D) structure was formed in extended Y6 crystal consisting of three kinds of Y6 molecular dimer (Fig. 1b). Note that due to the well-defined 3D molecular arrangement, an extra π–π interaction across four backbone plane occurs (as indicated by the green arrow in Fig. 1d), which is also observed from GIWAXS measurement of Y6-based thin films (discussed below). In contrast, such molecular packing structure is not found in IDT-based NFAs such as IT-4F or ITIC (Supplementary Fig. 6a), as discussed in previous reports^38^. Although molecular packing of IDT-based NFAs can be achieved via modulation of substituents of the end groups to form a 3D interpenetrating network structure, such as ITIC-2Cl-γ (Supplementary Fig. 6b)^39^, the packing between two closest molecules at a distance of ~3–4 Å only shows a simple overlap between the end groups, which represents the typical molecular packing observed for the conventional IDT-based NFAs. Such a molecular packing leads to small electronic couplings between adjacent molecules, which will limit the charge-transport properties. However, for Y6, the molecular packings are much more complex, as they consist not only of an overlap between end groups (Pair **1** and **2** shown in Fig. 1b) but also of an overlap between the cores (Pair **3**). The electronic couplings of the molecular pairs that define the main charge-transport pathways in the crystals were examined by first-principle quantum-mechanical calculations (see Methods for computational details). Such distinctive molecular packings in the Y6 crystal lead to much larger electronic couplings between two adjacent molecules. Our calculation results show that the maximum electronic coupling for electron and hole in the Y6 crystal is ~81 meV and 74 meV (Supplementary Table 4), respectively, much stronger than the IDT-based NFA systems, as discussed in previous reports^38^. As shown in Fig. 1e, the maximum electronic coupling for electron comes from the overlap between the LUMO wavefunctions mainly on the end groups (in Pair **1** and **2**), and that for holes from the overlap between the HOMO wavefunctions mainly on the DA′D cores (in Pair **3**), which leads to a 3D effective ambipolar transport network. This distinctive 3D effective hole transport network in acceptor may be favorable for effective hole transfer between donor and acceptor regardless of the acceptor molecular orientation at the donor/acceptor interfaces and can also minimize the hole transport distance in the acceptor domains for efficient hole transport, thus facilitating the overall hole transport in the BHJ blend (discussed below).

### Molecular packing in spin-coated films

**Fig. 2:**
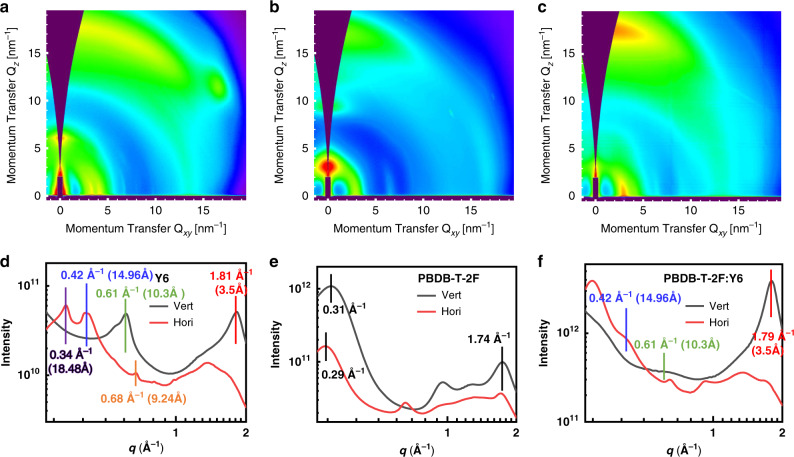
GIWAXS characterization of Y6 based systems. **a**–**f** Two-dimensional GIWAXS patterns (top) and profiles (bottom) of **a**, **d** the pristine Y6, **b**, **e** pristine PBDB-T-2F, and **c**, **f** PBDB-T-2F:Y6 films, respectively.

While we have revealed a distinctive packing structure in the Y6 single-crystal, it is important to evaluate whether or not this molecular packing is preserved in the spin-coated thin films of pristine Y6 and bulk-heterojunction blend involving the polymer donor (PBDB-T-2F). Figure 2a, b, d, e shows the GIWAXS measurements in pristine and blend films, respectively. As shown in Fig. 2a, d, we find that Y6 molecules in the pristine film adopt a face-on orientation to the substrate with a sharp out-of-plane π–π (010) peak at 1.81 Å^−1^ (*d* ~ 3.5 Å) (this is consistent with the π–π stacking distance obtained from the crystal structure, shown in red in Fig. 1d). Another sharp out-of-plane peak at 0.61 Å^−1^ (*d* ~ 10.3 Å) indicates the presence of longer-distance π–π stacking in the pristine Y6 film, suggesting more molecules stack regularly in the ***a*** direction (consistent with the longer π–π stacking distance across slide plane as obtained from the crystal structure, shown in green in Fig. 1d). Furthermore, the Y6 film shows regular molecular ordering in the in-plane direction, as evidenced by the presence of (100) and (200) lamellar peaks. The lamellar peaks at 0.34 Å^−1^ (*d* ~ 18.5 Å) and 0.42 Å^−1^ (*d* ~ 15.0 Å) are assigned to the lamellar distance between end-group stacking columns (blue) in the ***b*** direction and the adjacent end-group stacking columns (pink) in the ***c*** direction, determined as ~18.4 and 14.0 Å, respectively (see Fig. 1c, d and Supplementary Fig. 7b). The well-defined peak at 0.68 Å^−1^ (*d*  ~ 9.2 Å) in the in-plane direction is corresponding to backbone stacking in the ***b*** direction, determined as ~9.2 Å (see Fig. 1c and Supplementary Fig. 7a). The close agreement between the GIWAXS results and the single-crystal structure indicates that the crystalline nature of Y6 is preserved in the spin-coated film. In the blended film (Fig. 2c, f), Y6 maintains a distinctive molecular packing similar to that in the pristine film, as evidenced by the simultaneous presence of: (1) π–π diffraction peak at 1.79 Å^−1^ (*d* ~ 3.5 Å) along out-of-plane direction, (2) a backbone diffraction peak (though weaker than the pristine film) at 0.61 Å^−1^ along out-of-plane direction, and (3) a lamellar diffraction peak at 0.42 Å^−1^ in the in-plane direction, which are not found in the pristine PBDB-T-2F film (Fig. 2b, e).

### Molecular-dynamics simulations

**Fig. 3:**
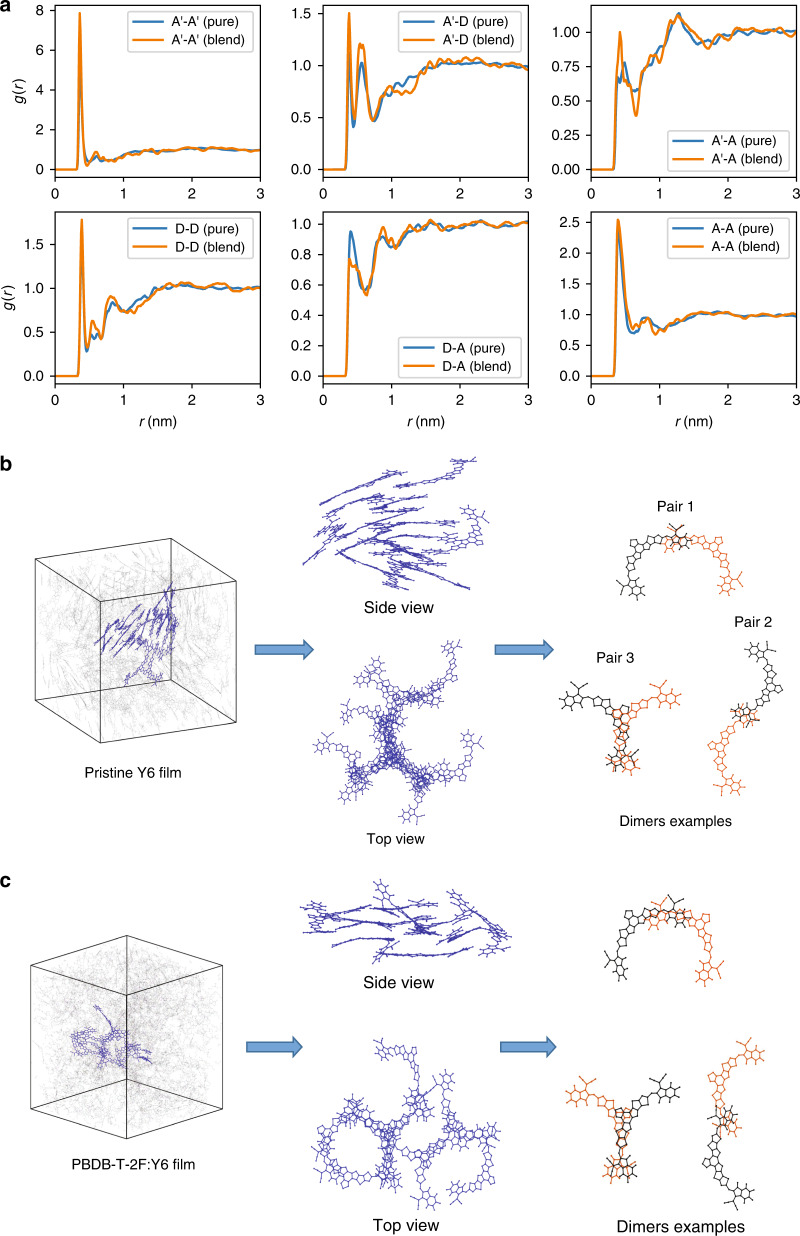
Molecular-dynamics (MD) simulations of Y6 based systems. **a**–**c** Radial distribution function (*g*) data for Y6 (**a**). The labels for the acceptor moieties are shown in Fig. 1a. The blue lines represent the data from the pristine acceptor films and the orange lines, the data from the donor/acceptor blends. Illustration of the molecular-dynamics simulations results for the packing in **b** the pristine Y6 and **c** PBDB-T-2F:Y6 films. A significant amount of Y6 dimers in both the pristine and blended films show similar packing compared to the Y6 crystal structures.

To further verify the molecular packings in the pristine and blend films, we carried out Molecular-dynamics (MD) simulations (see “Methods” for computational details). Figure 3a shows the radial distribution functions (RDFs), *g*(*r*), for both pristine and blend Y6 films (we note that the RDFs measure the probability of finding a particle some distance away from a reference particle, with a higher *g*(*r*) peak pointing to a larger packing density at a given distance). We find that the RDFs data of the pristine Y6 film are nearly the same as those of the Y6 blend, which indicates that the molecular packings of pristine Y6 are largely preserved in the blend. Furthermore, the RDF data suggest that all of the molecular fragments (A, D, and A′) exhibit a high first peak below 4 Å, implying strong π–π interactions among these molecular fragments. From snapshots of the MD simulations (Fig. 3b, c), a significant amount of the Y6 dimers in both the pristine and blended films show similar packing compared to the Y6 crystal structures (Fig. 1b). Importantly, our MD results are fully consistent with the GIWAXS results in both the pristine and blend films prepared by spin-coating. Here, it is worth noting that the remarkable aggregation of the Y6 molecule in the pristine and blend films is at some extent similar to those in fullerene-based materials^40,41^, which is not found in previous NFA-based materials.

As molecular packing plays a critical role in charge transport shown in our electronic coupling modeling above, we measured the electron and hole mobilities (*μ*_e_ and *μ*_h_) of the Y6 in electron- and hole- only devices using the space-charge-limited-current (SCLC) model (see “Methods” for details). We found that Y6 indeed show ambipolar charge transport properties with *μ*_e_ and *μ*_h_ values of 1.8 × 10^−4^ and 5.6 × 10^−4^ cm^2^ V^−1^ s^−1^, respectively (Supplementary Fig. 8), which is consistent with the 3D wavefunction delocalization shown above. These results may contribute both electron and hole transport in OSCs, which is rarely observed in NFAs. The capability for hole transport may also provide shortcut for hole transport across the gap of the polymer donor domains in the BHJ blend, because the HOMO offset between PBDB-T-2F and Y6 is very small (~0.05 eV) which makes the hole transfer between donor and acceptor possible^42^. Therefore, such distinctive ambipolar property of the acceptor will facilitate the overall hole transport in BHJ systems with small energy offsets for more efficient charge transport and collection.

### Exciton properties and voltage loss analysis

**Fig. 4:**
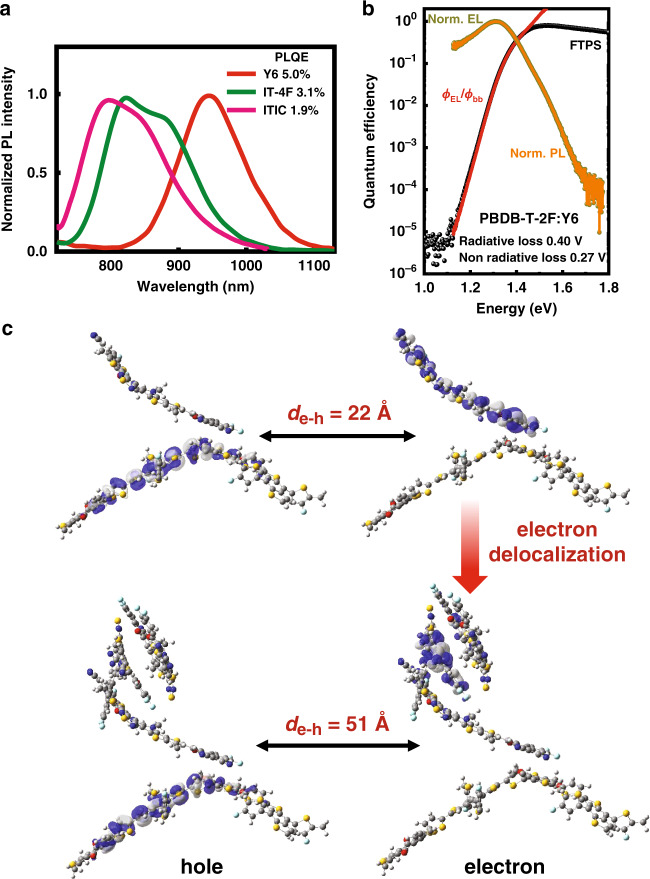
Charge recombination and separation properties. **a** Photoluminescence spectra of the Y6, IT-4F, and ITIC films excited at 660 nm together with their quantum efficiencies. **b** Semi-logarithmic plots of EQE evaluated by FTPS (EQE_FTPS_) (black spheres) and normalized EL (dark yellow spheres) as a function of energy for devices based on PBDB-T-2F:Y6. The ratio of *ϕ*_EL_/*ϕ*_bb_ was used to plot the EQE in the low energy regime (red line), where *ϕ*_EL_ and *ϕ*_bb_ represent the emitted photon flux and the room-temperature blackbody photon flux, respectively. The normalized PL spectra (orange lines) were measured based on the binary blend films. **c** Natural transition orbitals of the interfacial CT states by using the TD-*ω*B97XD/6-31G(d,p) method coupled with the PCM model for molecular clusters (left: one PBDB-T-2F donor fragment with one Y6 molecule; right: one PBDB-T-2F donor fragment with three Y6 molecules). Due to the delocalization of the electron wavefunction, the estimated distance (*d*_e–h_) between the hole and electron at the donor/acceptor interface increases from 22 Å for one Y6 molecule to 51 Å for clusters of three Y6 molecules.

The distinctive molecular packing in the Y6 crystal impact favorably not only the charge-transport properties but also the energy-transfer properties. Our calculation results show large exciton coupling between adjacent Y6 molecules in the range of 44–57 meV (see Supplementary Table 5). Applying a Marcus electron-transfer rate equation, we find that the hopping rate of excitons between adjacent Y6 molecules lies in the sub-picosecond range. Such large exciton coupling implies that the excitons are more delocalized in nature and can diffuse rapidly, and these characteristics generally lead to reduced exciton-vibration coupling and thus slower non-radiative decay rate (which generally scales positively with reducing optical gap^21^). We consider that this is reflected in the relatively high photoluminescence quantum efficiency (PLQE) of pristine Y6 film (~5%). This value is considerably higher than other IDT-based NFAs with similar optical gaps (below 1%, see ref. ^16^), and is comparable to IT-4F and ITIC which have significantly larger optical gaps (3.1% and 1.9%, respectively; see Fig. 4a). The reduced non-radiative decay rate is also reflected in the long exciton lifetime measured by time-resolved optical measurements (see Supplementary Fig. 9).

It was established that the ∆*V*_OC,nr_ can be minimized by: (1) reducing the energy offset between donor and acceptor to obtain similar energies of the local-exciton (LE) state and the CT state, and (2) enhancing the luminescent efficiency of the low-gap component in the blend^16^. Since we observe no clear sub-bandgap CT-state feature in both Fourier-transform photocurrent spectroscopies (FTPS) and electroluminescence (EL) measurement of the PBDB-T-2F:Y6 system (Fig. 4b), in the context of our recent three-state model^43^, it implies that the energy difference between the LE state and the CT state, Δ*G*_LE-CT_ (i.e., the driving force for exciton dissociation), must be very small, which is consistent with the very small ionization energy (HOMO) offset (0.05 eV) between donor and acceptor in the PBDB-T-2F:Y6 system. This feature allows hybridization of the CT state with the emissive LE state and thus increases the radiative rate of the CT state through an intensity borrowing mechanism^16^. Moreover, due to the small Δ*G*_LE-CT_, efficient transition from the CT state back to the LE state becomes energetically possible. We consider that the 3D hole transport network of Y6 clusters regardless of molecular orientation may also help to promote hole transfer between donor and acceptor at the interface. These properties allow the CT state to undergo radiative recombination via the emissive LE state. Together with the high PLQE of Y6, this explains the high Electroluminescence (EL) quantum efficiency (EQE_EL_ ~ 5.6 × 10^−3^%) of the PBDB-T-2F:Y6 device, leading to a Δ*V*_OC,nr_ of about 0.25 V according to the equation: Δ*V*_OC, nr_ = −(*kT/q*) ln(EQE_EL_) (see ref. ^44^) (Supplementary Fig. 10), which is very close to the value calculated from EL and FTPS measurements (Supplementary Table 2). Despite the significant improvement over traditional OSCs based on fullerene acceptors, we note that the performance of non-fullerene OSCs is still limited by the relatively large voltage losses of ~0.54 eV due to the relatively large ∆*V*_OC,nr_^16,45^. We believe that further improving the luminescent efficiency of OSC materials is crucial for achieving low voltage loss comparable with inorganic or perovskites photovoltaics (0.3–0.4 V; ∆*V*_OC,nr_ around 0.04–0.12 V)^24^.

### Electron delocalization promotes CT-state separation

More crucially, the delocalized nature of the electron wavefunction in Y6 clusters significantly improves the charge generation efficiency at the donor/acceptor interfaces. For example, as shown in Fig. 4c, due to the electron delocalization in a Y6 cluster (with three Y6 molecules, which were observed in above MD simulation), the estimated distance (*d*_e–h_) between the hole and electron at the donor/acceptor interface increases from 22 Å (for one Y6 molecule) to 51 Å. Due to this extended electron-hole separation, the Coulomb attraction of the interfacial electron-hole pair is reduced from 160 to 70 meV, implying a reduced energy barrier for the separation of the interfacial CT states into free charge. Although the driving force for exciton dissociation is small, the CT-state separation accelerated by the reduced Coulomb attraction competes with slow exciton dissociation (Supplementary Fig. 9c, d), which leads to large J_SC_ and FF in the device based on the PBDB-T-2F:Y6 blend. Barrierless free charge generation in Y6 system was observed in previous report by carrying out temperature-dependent measurements to reveal an exceptional small activation energy for CT-state separation of only 6 meV and efficient photocurrent generation down to *T* ≈ 100 K^34^. Therefore, we believe that weak Coulomb bound electron-hole pairs at the donor/acceptor interface in Y6 system could be one reason for that small activation energy for CT separation.

### High-performance OSC based on ternary system

Finally, as ternary blend is a quite widely used approach to further improve the OSC performance, we therefore also studied the molecular packing of Y6 in the ternary blend containing small amount of PC_71_BM acceptor and evaluate the device properties. By adding 20% weight content of PC_71_BM into PBDB-T-2F:Y6, it can improve the charge transport property and balance the electron/hole mobilities (Supplementary Fig. 11 and Table 6), which leads to a best device PCE of 16.5% with an enhanced FF (Supplementary Table 7 and 12a) while maintains similar charge generation efficiency (Supplementary Fig. 12b) and low voltage loss (Supplementary Fig. 13 and Table 8). A stabilized device with PCE of 15.63  ± 0.23% was certified by the National Renewable Energy Laboratory (NREL) using the asymptotic measurement method (Supplementary Fig. 14). Using GIWAXS measurements, we find that the addition of PC_71_BM molecules has little impact on the molecular packing of Y6, as the diffraction positions of the π-π peaks (at ~1.79 Å^−1^ and 0.61 Å^−1^ in the out-of-plane direction) and lamellar peaks (in-plane direction) remain largely unchanged (Supplementary Fig. 15). We therefore consider that the improved device PCE by the addition of PC_71_BM is mainly due to the improved charge transport property in the device.

## Discussion

In summary, we have reported a systematic study combining both experimental and theoretical modeling analyses to establish structure-property-performance relationships in state-of-the-art OSCs based on an efficient non-fullerene acceptor, Y6. We found that the curved geometry of the Y6 molecule leads to a distinctive π–π molecular packing, which was found in the single crystal and verified to preserve in the spin-coated films by GIWAXS measurement and MD calculation. Such molecular packing enables remarkable 3D delocalization of the electronic wavefunctions and the formation of effective electron and hole transport channels, making Y6 an ambipolar molecular material with balanced hole and electron transport properties. When it blends with the donor polymer (PBDB-T-2F) with a small HOMO energy offset, this distinctive feature could facilitate effective hole transfer and charge collection. More importantly, this unusual molecular packing plays a significant role in enabling both low voltage loss (mainly attribute to low ∆*V*_OC,nr_) and high charge generation efficiency of OSC blends comprising Y6 molecules. Firstly, the formation of delocalized excitons and low non-radiative decay rates in providing long exciton lifetime achieve high PLQE. In combination with a small energy difference between the exciton and CT states in the binary blend, the relatively low non-radiative voltage loss was achieved for such a low bandgap system. Secondly, the delocalization of electron wavefunctions at donor/acceptor interfaces significantly reduces the Coulomb attractions of hole and electron pairs, leading to reduced barrier for the CT-state dissociation. By integrating a ternary-blend strategy to improve charge transport without much affecting the Y6 molecular packing, a very high PCE of 16.5% is achieved with a enhance FF while maintains the low voltage loss and efficient charge generation. Our work highlights the fundamental structure-property-performance relationship of this state-of-the-art OSC system, thus pathing the way towards rational material design and the development of commercial OSC devices.

## Methods

### Materials

PBDB-T-2F (Mn = 37.0 kDa, Mw = 101.6 kDa, PDI = 2.74), IT-4F, ITIC, and [6,6]-Phenyl-C71-butyric acid methyl ester (PC_71_BM) were purchased from Solarmer Inc (China). Y6 was received from Prof. Zou Yingping and used without further purification. Diethyl zinc, 1.5 M solution in toluene, and tetrahydrofuran (THF) was purchased from Acros. MoO_3_, 1-chloronaphthalene (CN), and chloroform (CF) were purchased from Sigma-Aldrich. C_60_-SAM was purchased from 1-Material Inc. All other materials were purchased and used as received.

### Measurements and instruments

Cyclic voltammetry (CV) was carried out on a CHI660A electrochemical workstation with platinum electrodes at a scan rate of 100 mV s^−1^ against an Ag/Ag+ reference electrode with nitrogen-saturated solution of 0.1 M tetrabutylammonium hexafluorophosphate in acetonitrile. Potentials were referenced to the two ferrocenium/ferrocene couples by using ferrocene as an internal standard. UV-visible absorption spectra were measured using a HP 8453 spectrophotometer. PLQE of the films were recorded by a commercialized PLQY measurement system from Ocean Optics with excitation from a 660-nm LED.

### Single-crystal X-ray diffraction

Single crystals were grown through slow diffusion of ethanol (poor solvent) to its chloroform solution (good solvent). Suitable crystals were selected and recorded on Rigaku XtaLAB P2000 FR-X with a rotating copper anode and a Pilatus 200 K detector. The crystal was kept at 150 K during data collection. The structure was solved with the ShelXT program using intrinsic phasing and refined with the ShelXL refinement package using least squares minimisation.

### Fabrication and characterization of OSCs

The ITO glass substrates were cleaned sequentially under sonication with acetone, detergent, deionized water, and isopropyl alcohol and then dried at 60 °C in a baking oven overnight, followed by a 4-min oxygen plasma treatment. Then, a ZnO electron transport layer (a thickness of ~30 nm) was prepared by spin-coating at 5000 rpm 30 s from a ZnO precursor solution (diethyl zinc, 1.5 M solution in toluene, diluting in tetrahydrofuran) on ITO substrates, following by thermal annealing at 150 °C for 30 min. A C_60_-SAM monolayer was prepared by spin-coating C_60_-SAM solution (1 mg/ml, CB:THF = 2:1 by volume) at 4000 rpm 30 s, following thermal annealing at 100 °C for 5 min and then washed by CB:THF (2:1 by volume) solvent. The solutions of Y6 based systems (both binary and ternary blend solutions are using the weight ratio of donor to acceptor as 1:1) were prepared in CF (0.5% CN), respectively, and stirred on a hot plate at 50 °C over night. When the solutions cool down to room temperature, they were spin-coated on the pretreated substrates to obtain the thicknesses of ~100 nm by controlling the spinning rate. The Y6 based films were then annealed at 110 °C for 10 min, then were transferred to the vacuum chamber. At a vacuum level of 1 × 10^−7^ Torr, a thin layer (10 nm) of MoO_3_ was then thermally deposited as the anode interlayer, followed by thermal deposition of 100 nm of Ag as the top electrode through a shadow mask. The active area of all devices was 0.07 cm^2^. The *J*–*V* curves were measured in the forward direction from −0.2 to 1.2 V, with a scan step of 20 mV and a dwell time of 5 ms on a computer-controlled Keithley 2400 source meter under 1 sun, the AM 1.5 G spectra came from a class solar simulator (Enlitech, Taiwan), and the light intensity was 100 mW cm^−2^ as calibrated by a China General Certification Center-certified reference monocrystal silicon cell with KG1 filter (Enlitech). Before the *J*–*V* test, a physical mask with an aperture with area of 0.04 cm^2^ (determined by image measuring instrument) was used to define the device area. All the above measurements were performed in glove box at room temperature. The EQE spectra measurements were performed on a commercial EQE measurement system (QE-R3011, Enlitech). The IQE of the device was the measured EQE divided by the BHJ absorption which is calculated through the transfer matrix method (TMM) based optical simulation^46^. The optical parameters of *n* and *k* for different films were measured using a dual rotating-compensator Mueller matrix ellipsometer (ME-L ellipsometer, Wuhan Eoptics Technology Co., Wuhan, China). The probed wavelength range of Psi and Delta is 350–1650 nm. In general, we first use Cauchy model to fit the transparent region of organic semiconductor. Then, Bspline is used for the remaining part. After that, the parameterization is performed with combinations of Tauc-Lorentz model and Gaussian model. Finally, film thickness, incidence angle, roughness, and all model parameters are optimized simultaneously for a small enough mean-squared error^47^.The transient photocurrent and transient photovoltage of the devices were were carried out with Paios 4.0 Measurement Instrument (FLUXiM AG, Switzerland). Photostability testing of the OSCs were carried out in a home-built measurement system with the help of Guangzhou Crysco Equipment Company Limited. The OSCs were encapsulated with cover glass and then transferred to a light-soaking degradation setup for photoaging test in ambient condition. The *J*–V characteristics of the studied devices were probed continuously under illumination by a light emitting diode (LED) light source irradiating at a wide wavelength range of 360–960 nm of the LED was set to produce the same *J*_SC_ as under an AM1.5 G solar simulator.

### Fabrication and characterization of electron- and hole-only devices

Devices were fabricated to measure the electron and hole mobility by using the SCLC method. The electron-only device structure was ITO/Al/ pristine or blend films/LiF/Al; The hole-only device structure was ITO/PEDOT:PSS/pristine or blend films/MoO_3_/Ag. The mobility was determined by fitting the dark current to the model of a single-carrier SCLC, which is described by the equation *J* = (9/8)*ε*_0_*ε*_r_*μ*((*V*^2^)/(*d*^3^)), where J is the current density, *μ* is the zero-field mobility, *ε*_0_ is the permittivity of free space, *ε*_r_ is the relative permittivity of the material, *d* is the thickness of the active layers, and *V* is the bias voltage. The mobility can be calculated from the slope of the *J*^1/2^–*V* curves.

### Time-resolved photoluminescence spectroscopy

The encapsulated thin-film samples were photoexcited using a Ti:sapphire femtosecond oscillator tuned to 710 nm with a repetition rate of 76 MHz. The photoluminescence was collected by an Acton Spectrapro 275 spectrometer equipped with a Becker and Heckl single photon counter using time resolved single photon correlation to obtain time resolved PL.

### Transient absorption spectroscopy

The probe pulse was a white light continuum generated by focusing the 800 nm output of the Ti:Sapphire laser amplifier (Coherent Legend Elite 1 kHz, 150 fs) onto a sapphire plate. The 860 nm pump pulse was generated in a commercial optical parametric amplifier (Light conversion OPerA SOLO) and was chopped at 500 Hz and polarised to the magic angle with respect to the probe. The optical pulses were spatially overlapped in the encapsulated thin-film sample and temporally delayed using a motorised delay stage. The probe was guided into a spectrometer (acton spectrapro 275) equipped with a Si photodiode array detector and we record the pump-induced change in transmission (∆*T*/*T*). The pump fluence was set at ~2 µJ cm^−2^ per pulse.

### EL and FTPS measurements

Electroluminescence measurements were performed by applying an external voltage/current source through the devices. The luminescence spectra were collected in a back-scattering geometry, dispersed by an iHR320 monochromator (Horiba Jobin-Yvon) and recorded with a Peltier-cooled Si CCD (Synapse, Horiba Jobin-Yvon). The FTPS measurements were carried out using a Bruker Vertex 70 Fourier-transform infrared (FTIR) spectrometer, equipped with a quartz tungsten halogen lamp and a quartz beamsplitter as well as an external detector option. A low-noise current amplifier (Femto DLPCA-200) was used to amplify the photocurrent produced on illumination of the photovoltaic devices with light modulated by the FTIR. The output voltage of the current amplifier was fed back into the external detector port of the FTIR. Absolute EQE photovoltaic values were redrawn by correcting the FTPS to the EQE of the corresponding solar cells.

### Characterization of GIWAXS

The GIWAXS measurement was performed at the small-angle and wide-angle X-ray scattering beamline at the Australian Synchrotron. A Pilatus 1 M two-dimensional detector with 0.172 mm × 0.172 mm active pixels was used in integration mode. The detector was positioned ~300 mm downstream of the sample location. The precise sample-to-detector distance was determined with a silver behenate standard. An 11 KeV incident X-ray with a 0.25 mm × 0.1 mm spot was used to provide a sufficiently large q space. The two-dimensional raw data were reduced and analyzed with a modified version of Nika. The presented GIWAXS patterns were corrected to represent real *Q*z and *Q*xy axes with the consideration of the missing wedge. The critical incident angle was determined by the maximized scattering intensity of sample scattering with a negligible contribution of underneath layer scattering. The shallow incident angle scattering was collected at 0.02°, which rendered the incident X-ray an evanescent wave along the top surface of the thin films.

### Theoretical simulations

For the Y6 isolated molecules, the ground-state (S_0_) geometries were initially optimized with the range-separated functional *ω*B97XD (with the range-separation parameter *ω* set initially at the default value of 0.2 bohr^−1^) and the 6-31 G(d,p) basis set^48^. Then, following our earlier investigations^49^, an iteration procedure was employed to non-empirically tune the *ω* parameters with the implicit consideration of the dielectric environment via the polarizable continuum model (PCM); the dielectric constant *ε* was chosen to be 3.5, a representative value of organic semiconductor materials. Based on these non-empirically tuned *ω* parameters, the geometries of the cation and anion species were optimized, and the Marcus reorganization energies were derived from the adiabatic potential energy surfaces of the neutral and charged states of the molecule. All the excited-state properties of the molecules were examined at the TD tuned-*ω*B97XD/6-31 G(d,p) level of theory. Natural Transition Orbital (NTO)^50^ analyses were also performed to characterize the nature of the electronic states. To reduce the computational cost while maintaining the reliability of our results, all the alkyl chains were replaced with methyl groups. The molecular pairs with the shortest distances were extracted from the Y6 crystal to estimate the electronic couplings for hole and electron transport via the fragment orbital approach^51^; the results are listed in Supplementary Table 4. In addition, the electronic couplings between the first singlet excited states, related to exciton diffusion, were estimated via the approach developed by Mennucci and co-workers^52^; the results are listed in Supplementary Table 5. The periodic DFT calculations on the Y6 crystal were performed with the Vienna Ab initio Simulation Package (VASP)^53^, using the PBE0 hybrid exchange-correlation functional. The plane-wave energy cutoff was set as 400 eV. A 3 × 3 × 1 k-mesh sampling in the Monkhorst-Pack scheme was used. The wavefunctions for the valence band maximum (VBM) and conduction band minimum (CBM) were visualized by the VESTA software^54^.

The MD simulations of the pristine Y6 film, PBDB-T-2F:Y6 blends were performed in LAMMPS package^55^. The OPLS-AA force field was used to describe the bonded and non-bonded potentials^56^. In addition, the atomic partial charges, equilibrium bond lengths, equilibrium bond angles, and dihedral potentials were re-parameterized based on our DFT calculations. The atomic partial charges were calculated at the long-range corrected *ω*B97XD/cc-PVTZ level of theory^57^, and fitted using the restrained electrostatic potential (RESP) method by AmberTools. The equilibrium bond lengths and angles were updated based on *ω*B97XD/6-31 G(d,p) optimized geometries. The dihedral potentials that dictate the planarity of the conjugated molecules were re-parameterized based on the DFT potential energy surfaces. All of the DFT calculations were performed using Gaussian 16 package^58^.

For pristine films, 200 acceptor molecules were used in the simulation. For blends, the polymer to acceptor ratio was kept at 1:1 by weight. We used 10 chains of PBDB-T-2F each with 20 monomers for all of the blends. The bulk structures were constructed using the following procedure. First, the molecules were randomly placed into a cubic box under periodic boundary condition at low density. Then, the NPT-MD was performed for 30 ns at 650 K to equilibrate the structures, and subsequently cooled to 300 K at a rate of 10 K ns^−1^. Lastly, another NPT-MD was performed at 300 K for 30 ns in order to obtain the final structures. All of the MD simulations employed velocity-Verlet integrator at 2.0 fs time step with SHAKE algorithm. The temperature and pressure were controlled using Nose-Hoover thermostat and barostat, respectively. The cutoff for nonbonded interactions was set to 12 Å, and the electrostatics was solved using the particle-particle particle-mesh method. All of the results were analyzed from the last 10 ns of the MD simulations and averaged from three independent boxes.

## Supplementary information

Supplementary Information

## Data Availability

The data supporting the results of this work are available from the corresponding authors upon reasonable request. The supplementary crystallographic data for this work could be checked in The Cambridge Crystallographic Data Centre (CCDC) via www.ccdc.cam.ac.uk/data_request/cif by the series number as 2015912.
