# Supplementary material for: Delocalization of exciton and electron wavefunction in non-fullerene acceptor molecules enables efficient organic solar cells
Source: Nat Commun. 2020 Aug 7;11:3943. doi: 10.1038/s41467-020-17867-1 (PMC7414148; doi:10.1038/s41467-020-17867-1)
Supplement: Supplementary file 1 — Supplementary Information [file 41467_2020_17867_MOESM1_ESM.pdf]

Supplementary Information

Delocalization of exciton and electron wavefunction in non-fullerene acceptor molecules enables efficient organic solar cells

Zhang et al.

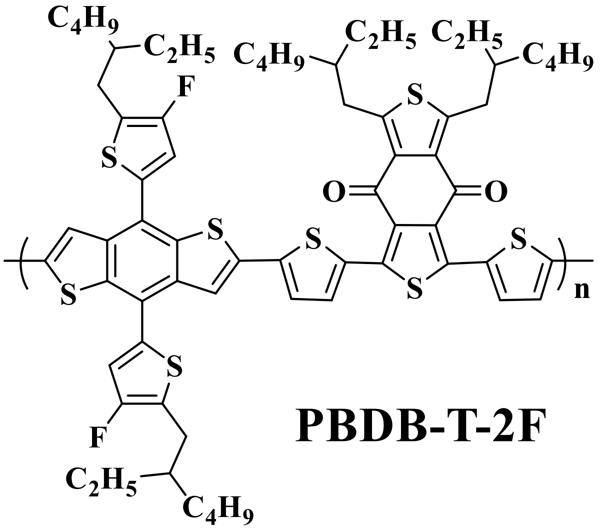

Supplementary Figure 1 | Molecular structure of the donor polymer (PBDB-T-2F).

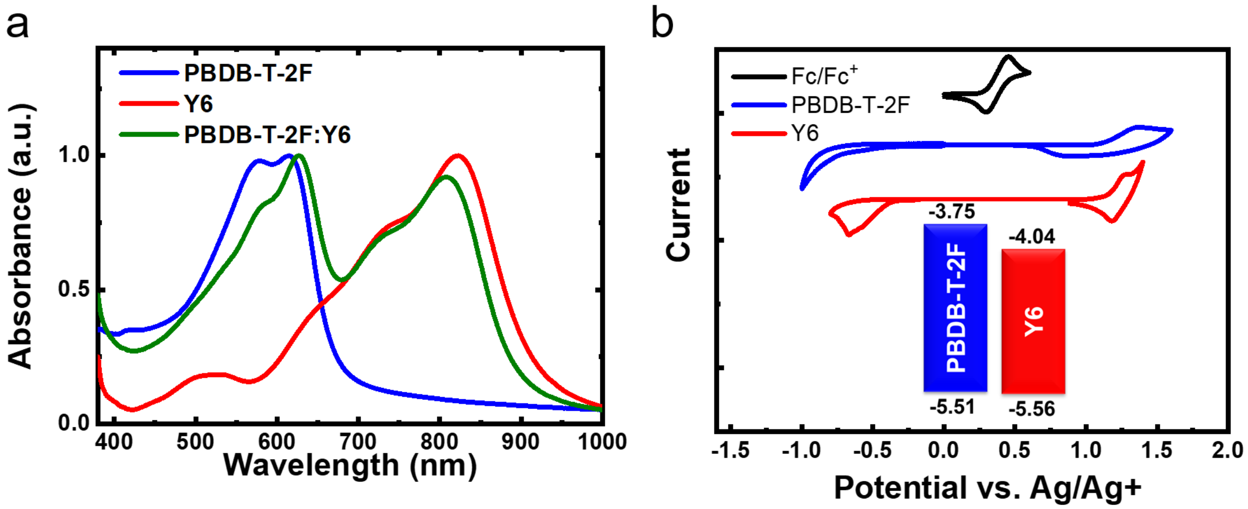

Supplementary Figure 2 | Absorption spectra and cyclic voltammetry (CV) measurements. (a) Absorption spectra of the pristine PBDB-T-2F, Y6, and PBDB-T-2F:Y6 blend films. (b) CV and energy levels (the insert) of PBDB-T-2F and Y6 films on ITO glass in 0.1 mol L^{-1} Bu_4NPF_6 in acetonitrile at a scan rate of 100 mV s^{-1}.

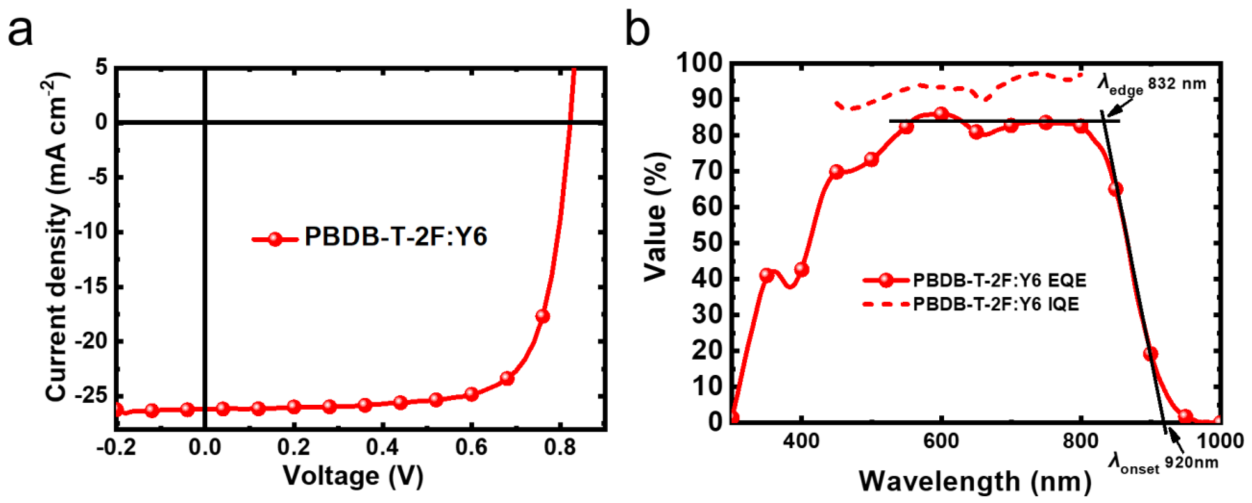

Supplementary Figure 3 | OSC photovoltaic performance. (a) J-V curves and (b) EQE and IQE spectra of the Y6 based device.

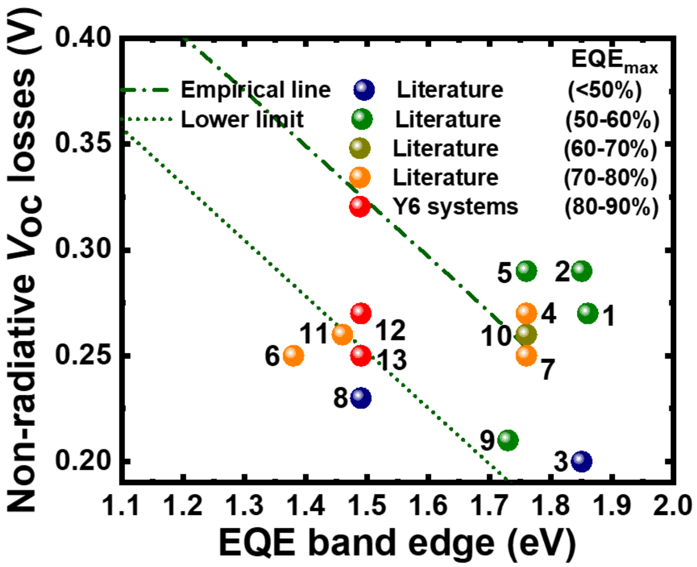

Supplementary Figure 4 | Summary of the OSCs with low Non-radiative V_{OC} losses (< 0.30 eV). $\Delta V_{OC, nr}$ versus EQE band edges for reported NFA OSCs (see Supplementary Table 3 for details) and the systems in the present work, together with EQE_{max} values. The empirical and lower limit lines are extracted from reference[1].

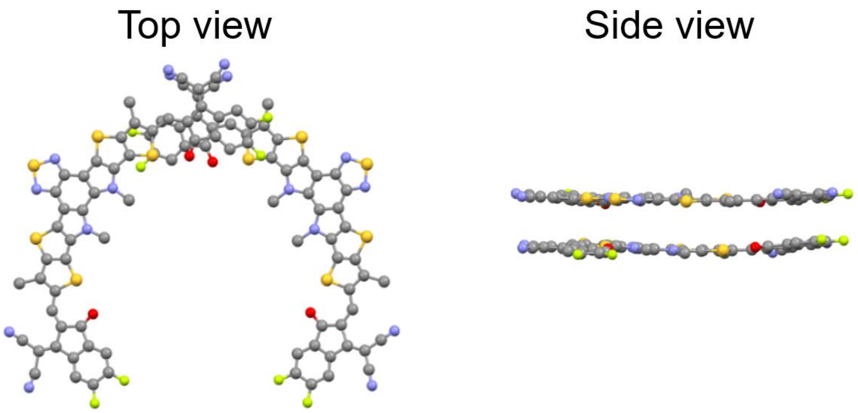

Supplementary Figure 5 | Single-crystal structures of Y6. Top (left) and side (right) views of minimum repeat unit.

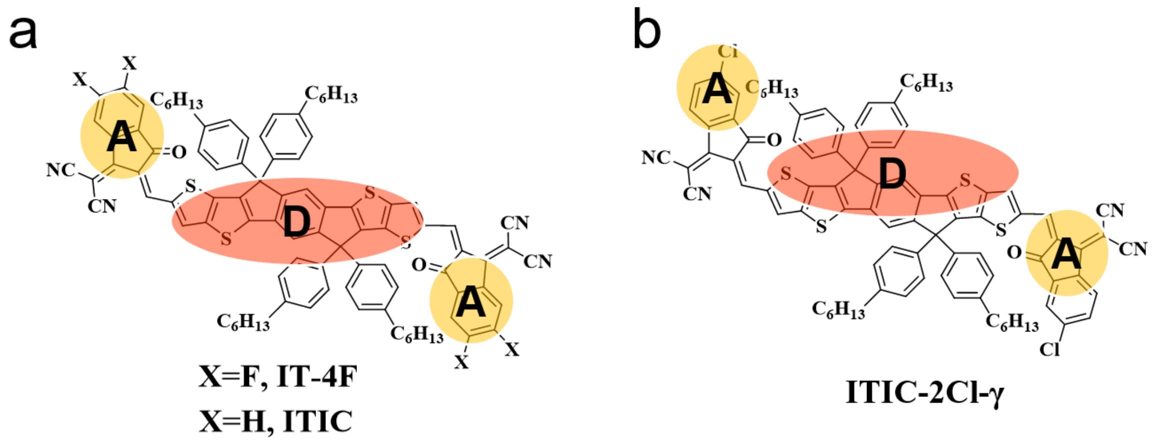

Supplementary Figure 6 | Molecular structure of the IDT-based NFAs. a, IT-4F and b, ITIC-2Cl-γ.

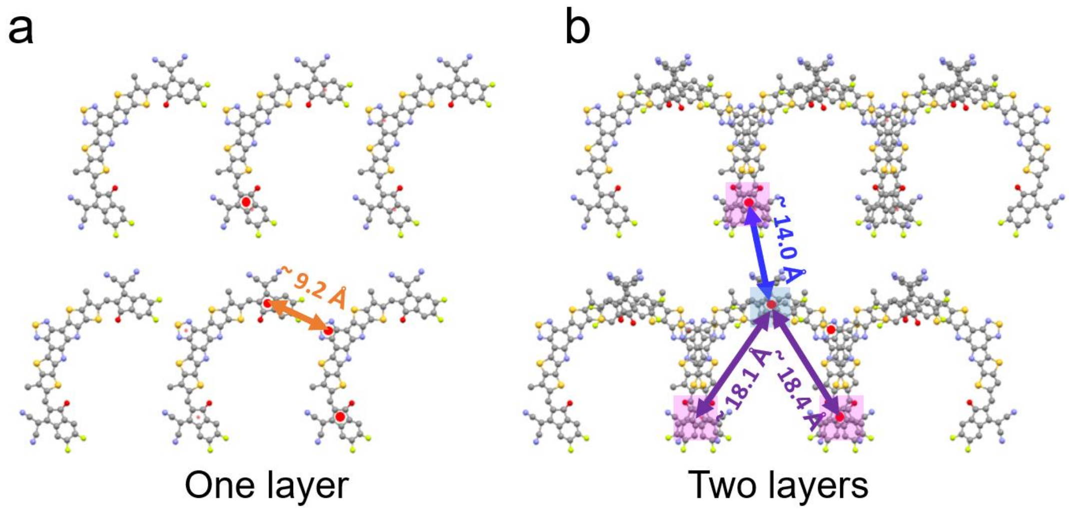

Supplementary Figure 7 | Crystal structure of Y6 with (a) one layer and (b) two layer.

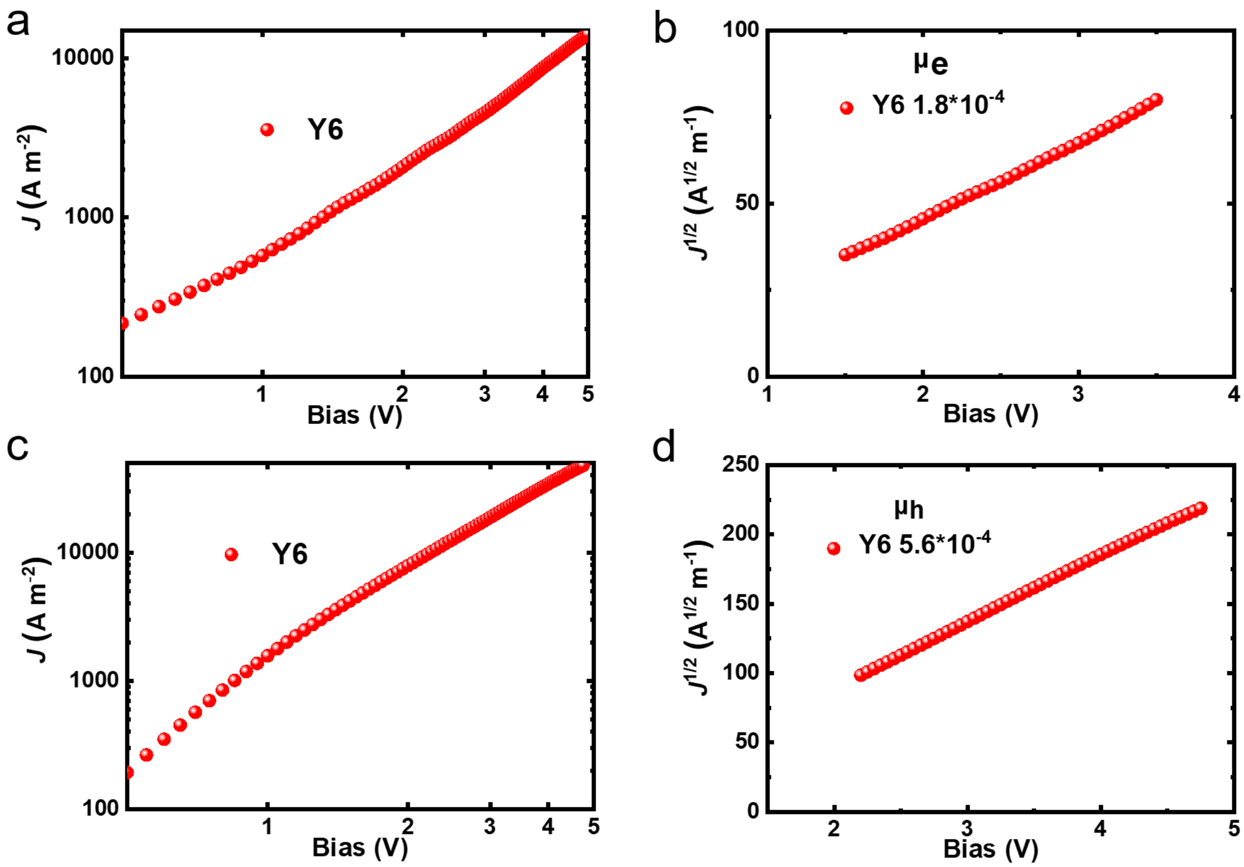

Supplementary Figure 8 | SCLC electron and hole mobility. Dark *J*–*V* and $J^{1/2}$–*V* plots for single-electron (a, b) and single-hole (c, d) devices based on pristine Y6 (~100 nm) film.

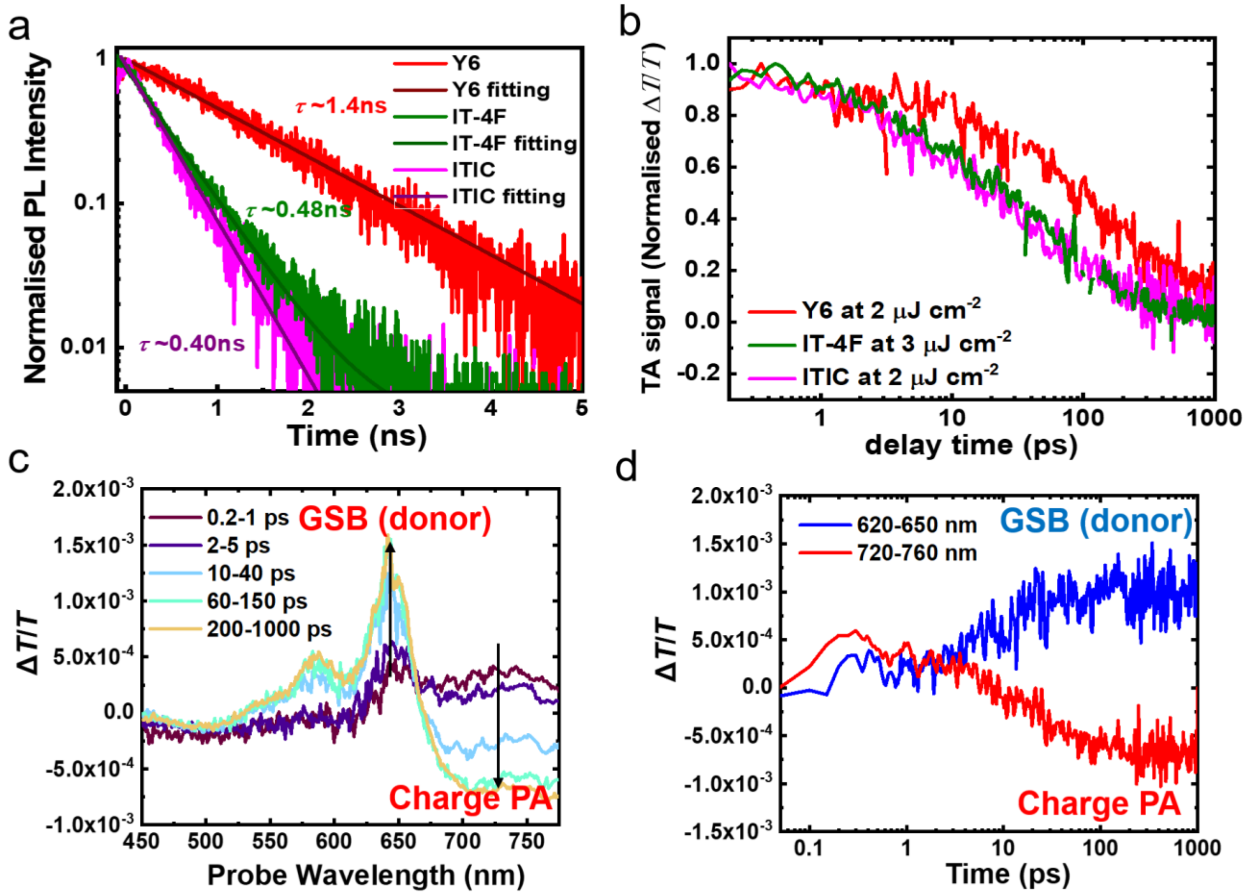

Supplementary Figure 9 | Exciton decay dynamics. a. Time-resolved photoluminescence decay of the pristine Y6, IT-4F, and ITIC films. b. Transient absorption signal of the exciton versus time in the pristine Y6, IT-4F, and ITIC films pumped at ~ 600 nm, and probed by integrating between 840-870, 750-780, and 725-755 nm, respectively. c and d. TA spectra and signal of the PBDB-T-2F:Y6 blend films excited at 860 nm.

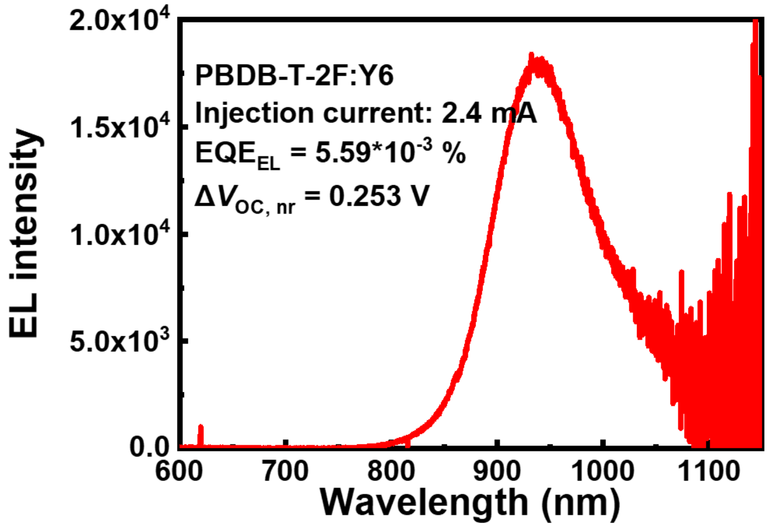

Supplementary Figure 10 | EQE$_{EL}$ spectra of the OSC based on PBDB-T-2F:Y6 system.

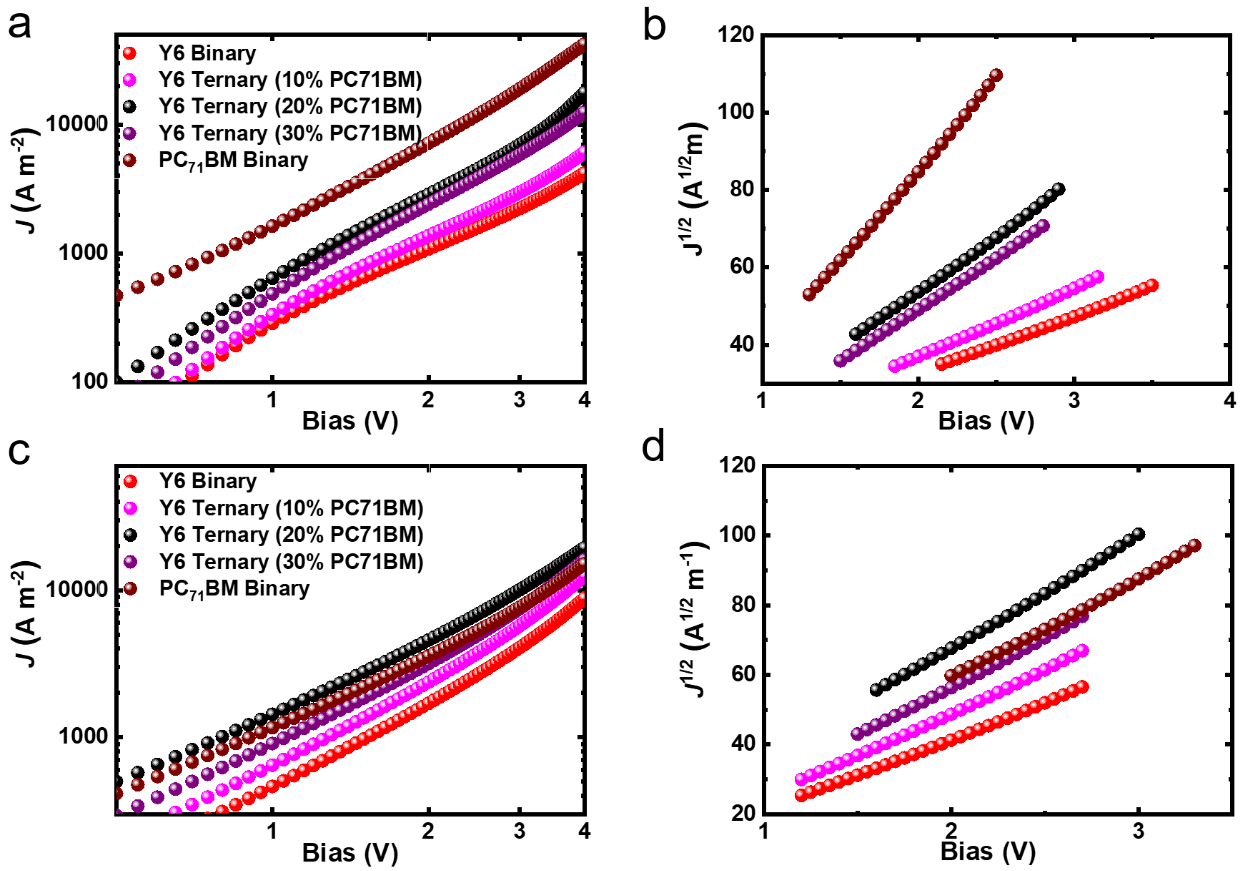

Supplementary Figure 11 | SCLC electron and hole mobilities. Dark J–V and $J^{1/2}$–V plots for single-electron (a, b) and single-hole (c, d) devices based on the Y6 binary and ternary films (~100 nm).

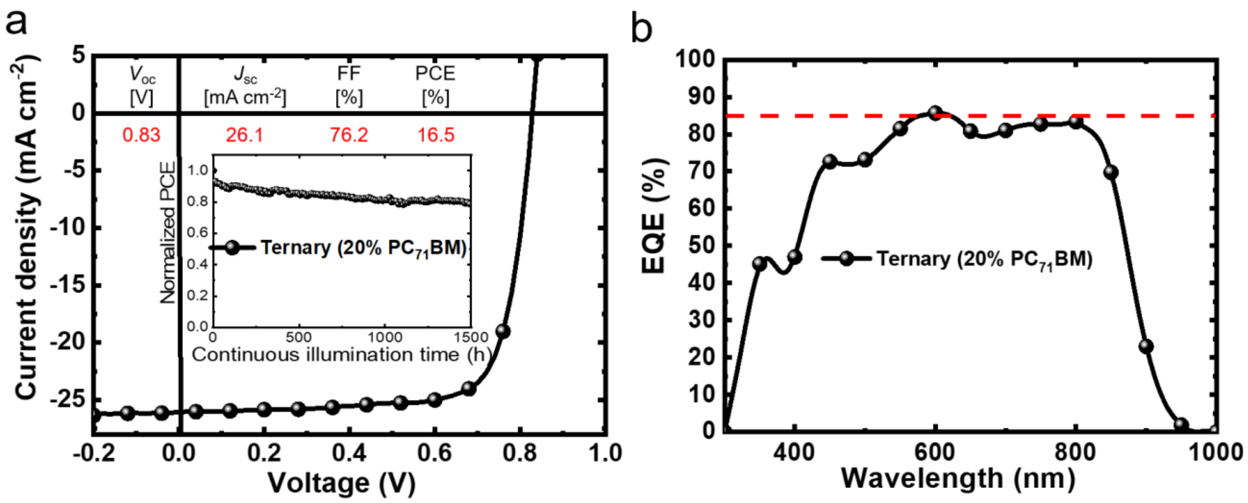

Supplementary Figure 12 | Device performance of the Y6 ternary OSC. (a) *J*-*V* curve of the Y6 ternary device (20% $PC_{71}BM$). The inset shows the variation of normalized PCE of this ternary device with testing time under continuous illumination of a LED light (from 360 to 960 nm) in air with encapsulation. (b) EQE spectrum of the Y6 ternary device (20% $PC_{71}BM$) (the integrated *J*sc from the EQE spectrum is 25.4 mA/cm^2, with an error < 5% compared to the *J*sc obtained from the J-V scan in Supplementary Table 6).

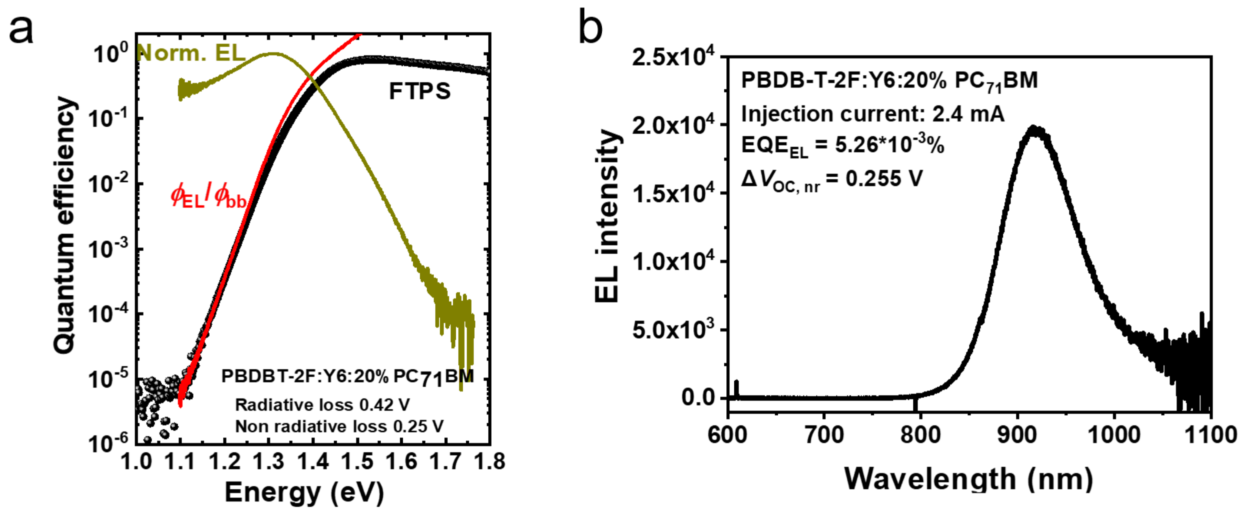

Supplementary Figure 13 | Voltage loss analysis. (a) Normalized EL, ϕEL/ϕbb, EQE_{FTPS}, and (b) EQE_{EL} spectra for the PBDB-T-2F:Y6:20% $PC_{71}BM$ ternary device.

NREL
NATIONAL RENEWABLE ENERGY LABORATORY

Photovoltaic Cell Data Compilation

Calibration Conducted For:

Hen-Hsin Wu
Enli Technology Co. Ltd.
1F., No.96, Luke 5th Rd., Luzhu Dist.
Kaohsiung City 821, Taiwan

Comments

Current vs. voltage was measured using our asymptotic Pmax method and using our standard IV sweep method.

Asymptotic Method
Asymptotic scans are measured by holding the cell at a set of voltages near Vmax until the current reaches an asymptotic level for each voltage. The voltage vs. asymptotic current data is then applied to our standard Pmax derivation algorithm.

Standard Sweep
Fast scans from forward to reverse and from reverse to forward before and after the asymptotic Pmax measurement indicated that this device was not susceptible to some commonly seen measurement artifacts for OPV. Consequently, we have included standard IV sweeps in this data compilation.

Data Collected By:

National Renewable Energy Laboratory
Solar Cell/Module Performance Group
15013 Denver West Parkway
Golden, CO 80401-3305

Contents

Y-OPV-01_middle<br>HLBQE 181116-122458	QE	3
Y-OPV-01_middle<br>X25 LIV 181115-172622 SMU	LIV	4
Y-OPV-01_middle<br>X25 LIV 181115-175149	LIV	5
Y-OPV-01_middle<br>X25 LIV 181115-175723	LIV	6

South China University of Technology
OPV

Sample: Y-OPV-01_middle | Temperature = 24.7 ± 2°C
Nov 16, 2018 12:24 | Device Area = 0.04113 cm^2

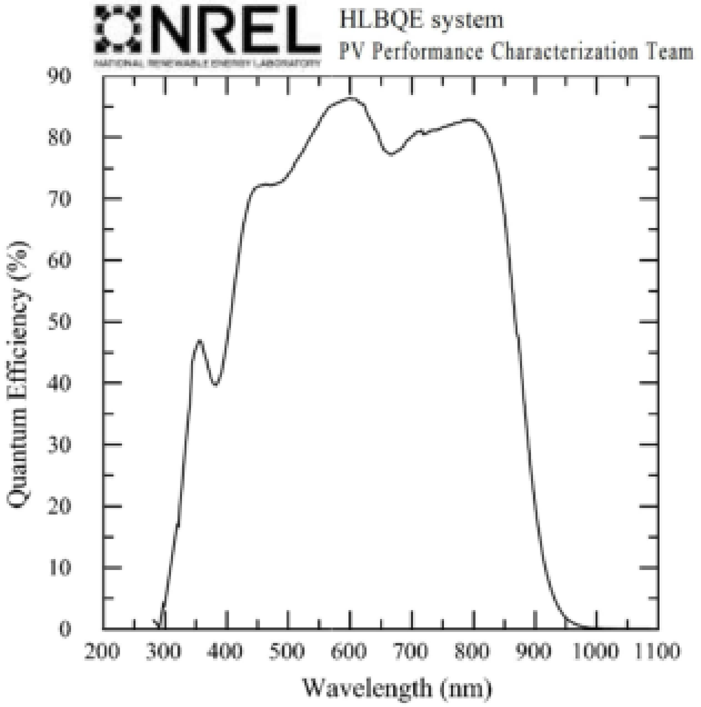

zero voltage bias
Light Bias = 0.320 mA into 0.041 cm^2

Unfiltered bias light

Rapid Scan

South China University of Technology

OPV Cell

Device ID: Y-OPV-01_middle
Nov 15, 2018 17:26
Spectrum: ASTM G173 global

Device Temperature: 25.0 ± 3.5 °C
Device Area: 0.04113 ± 0.4% cm^2
Irradiance: 1000.0 W/ m^2

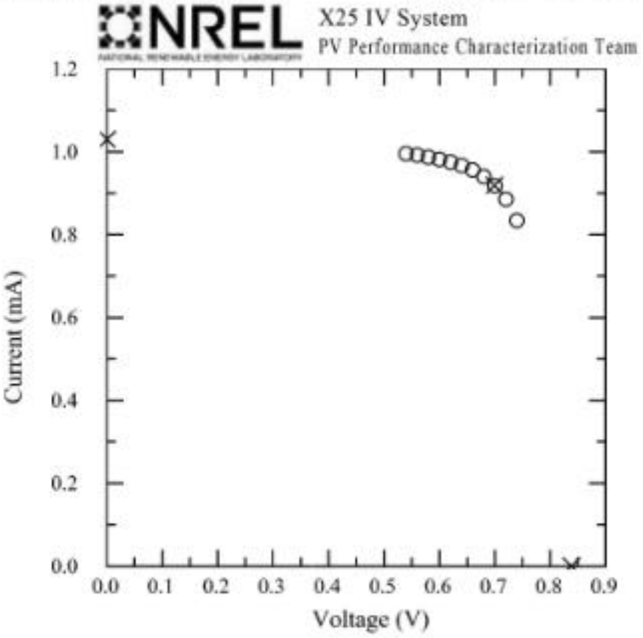

V_{oc} = 0.8381 ± 0.0092 V
I_{sc} = 1.030 ± 0.011 mA
J_{sc} = 25.03 ± 0.33 mA/cm^2
Fill Factor = 74.47 ± 0.56 %

I_{max} = 0.919 ± 0.010 mA
V_{max} = 0.6994 ± .0021 V
P_{max} = 0.6427 ± 0.0084 mW
Efficiency = 15.63 ± 0.23 %

Asymptotic method

South China University of Technology
OPV Cell

Device ID: Y-OPV-01_middle
Nov 15, 2018 17:51
Spectrum: ASTM G173 global

Device Temperature: 25.0 ± 1.5 °C
Device Area: 0.04113 cm^2 ± 0.4 %
Irradiance: 1000.0 W/m^2

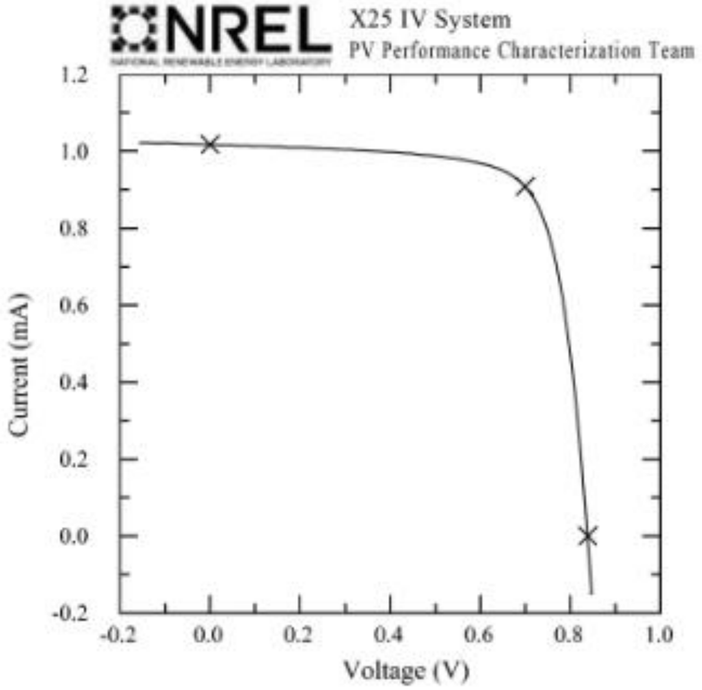

V_{oc} = 0.8388 ± 0.0029 V
I_{sc} = 1.0175 ± 0.0065 mA
J_{sc} = 24.74 ± 0.19 mA/cm^2
Fill Factor = 74.39 ± 0.29 %

I_{max} = 0.9081 ± 0.0057 mA
V_{max} = 0.699080 ± 0.000098 V
P_{max} = 0.6349 ± 0.0040 mW
Efficiency = 15.43 ± 0.12 %

Forward to reverse, using standard method.

page 6

South China University of Technology

OPV Cell

Device ID: Y-OPV-01_middle | Device Temperature: 25.0 ± 1.5 °C
Nov 15, 2018 17:57 | Device Area: 0.04113 cm^2 ± 0.4 %
Spectrum: ASTM G173 global | Irradiance: 1000.0 W/m^2

NREL
X25 IV System
PV Performance Characterization Team

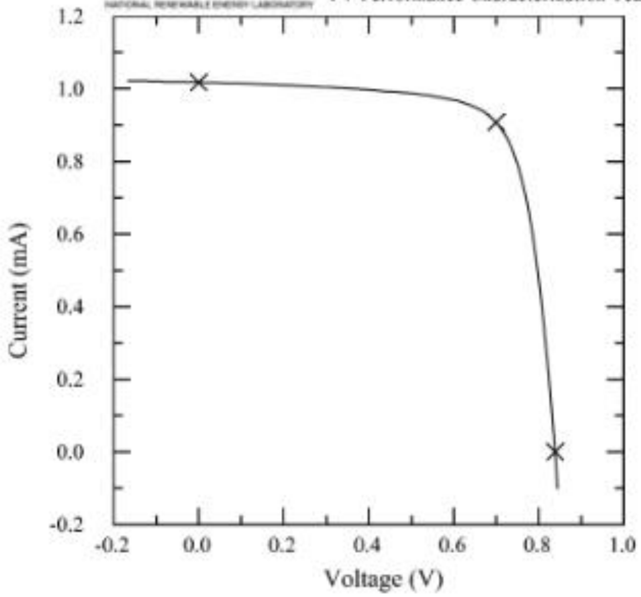

V_{oc} = 0.8388 ± 0.0028 V | I_{max} = 0.9074 ± 0.0058 mA
I_{sc} = 1.0178 ± 0.0065 mA | V_{max} = 0.699494 ± 0.000098 V
J_{sc} = 24.74 ± 0.19 mA/cm^2 | P_{max} = 0.6347 ± 0.0040 mW
Fill Factor = 74.35 ± 0.28 % | Efficiency = 15.43 ± 0.12 %

reverse to forward, using standard method

Supplementary Figure 14 | Certification report from NREL. EQE spectrum, J-V curve tested by using the asymptotic method and standard methods (forward to reverse and reverse to forward).

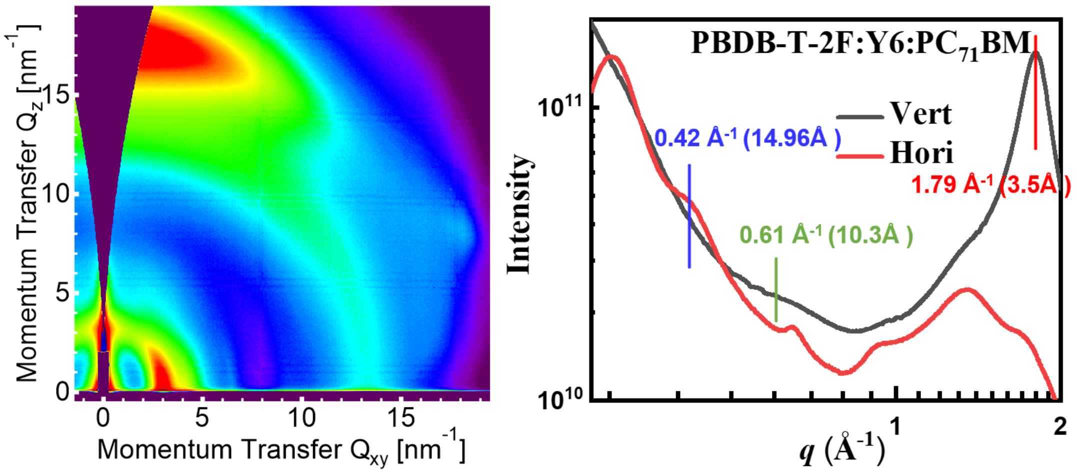

Supplementary Figure 15 | Morphology characterization. Two-dimensional GIWAXS patterns (left) and profiles (right) of theY6 ternary blend films.

Supplementary Table 1 | Photovoltaic performance of the Y6 based devices.

**Device**[e]	V_{oc} **[V]**[a]	E_g^{onset} **(eV)**[b]	J_{sc} **[mA cm^{-2}]**[a]	**FF [%]**[a]	**PCE [%]**[a]	**Voltage loss (V)**[c]	**EQE$_{max, acceptor}$ (%)**[d]
PBDB-T-2F:Y6	0.82±0.01	1.35	25.9±0.2	73.3±0.4	15.7±0.1	0.53	84

[a]Statistical data obtained from 10 independent devices under AM 1.5 G illumination at 100 mW cm^{-2}. [b]E_{opt}^{onset} = 1240/λ_{onset} was determined from the onset of EQE spectra (Supplementary Fig. 3b). [c]The voltage loss was calculated by subtracting V_{oc} from E_{opt}^{onset}/q. [d]The device architecture is ITO/ZnO/C_{60}-SAM/Active layers/MoO_3/Ag.

Supplementary Table 2 | Quantification of voltage losses in the Y6 based device.

Device	E_{opt}^{edge} (eV)[a]	$V_{OC, rad}$ (V)[b]	$\Delta V_{OC, rad}$ (V)[c]	V_{OC} (V)	$E_{opt}^{edge}/q - V_{OC}$ (V)	$\Delta V_{OC, nr}$ (V)[d]
PBDB-T-2F:Y6	1.49	1.09	0.40	0.82	0.67	0.27

[a]E_{opt}^{edge} was determined from the intersection of the EQE edge and the local EQE maximum (Supplementary Fig. 3b)[2]. [b]$V_{OC, rad}$ was calculated from EL and FTPS measurements (Fig. 4b) by using the following equation: $V_{OC, rad} = kT/q \ln(J_{SC}/J_{0, rad} + 1)$, where $J_{0, rad} = q \int_0^{\infty} EQE \phi_{bb} dE$ [3]. [c] $\Delta V_{OC, rad} = E_{opt}^{edge} - V_{OC, rad}$. [d]$\Delta V_{OC, nr} = V_{OC, rad} - V_{OC}$.

Supplementary Table 3 | Summary of NFA OSC systems with a non-radiative voltage loss < 0.3 V and different E_{opt}^{edge} and EQE_{max}.

Number	Device	E_{opt}^{edge} (eV)	$\Delta V_{OC, nr}$ (V)	EQE_{max} (%)	Ref.
1	BDT-ffBx-DT:PDI6	1.86	0.27	52	4
2	BDT-ffBx-DT:PDI4	1.85	0.29	55	4
3	BDT-ffBx-DT:SFPDI	1.85	0.20	47	4
4	PffBT4T-2DT:IDTBR	1.76	0.27	76	5
5	PffBT4T-2DT:FBR	1.76	0.29	58	5
6	PBQ-QF:IEICO-4F	1.38	0.25	70	6
7	PvBDTTAZ:O-IDTBR	1.76	0.25	71	6
8	PTB7-Th:IEICO	1.49	0.23	47	6
9	PDCBT-2F:IT-M	1.73	0.21	50	6
10	P3TEA:SF-PDI2	1.76	0.26	66	7
11	PBDB-T:Y2	1.46	0.26	73	8
12	PBDB-T-2F:Y6	1.49	0.27	85	This work
13	PBDB-T-2F:Y6:$PC_{71}BM$	1.49	0.25	85	This work

Supplementary Table 4 | Electronic couplings (V) and charge-transfer rates (k) of the molecular pairs extracted from the Y6 crystal for hole and electron transport. The molecular pairs are depicted in Fig. 1b in the main text.

	V_{hole} /cm^{-1} [meV]	$k_{hole\text{-}transfer}$ (s^{-1})	$V_{electron}$ /cm^{-1} [meV]	$k_{electron\text{-}transfer}$ (s^{-1})
Y6: pair 1	142 [18]	1.5×10^{12}	652 [81]	3.9×10^{13}
Y6: pair 2	17 [2]	2.1×10^{10}	-175 [-22]	2.8×10^{12}
Y6: pair 3	600 [74]	2.6×10^{13}	549 [68]	2.8×10^{13}

Supplementary Table 5 | Exciton couplings in the first singlet excited state (S_1) of the molecular pairs extracted from the Y6 and IT-4F crystals. The molecular pairs are depicted in Fig. 1b in the main text.

	**Exciton Coupling /cm^{-1} [meV]**
Y6: pair 1	403 [50]
Y6: pair 2	-460 [-57]
Y6: pair 3	355 [44]

Supplementary Table 6 | Summary of hole and electron mobilities of the blend films evaluated by SCLC method.

**Blend films**	**Electron mobility (10^{-4} cm^2 V^{-1} s^{-1})**	**Hole mobility (10^{-4} cm^2 V^{-1} s^{-1})**
PBDB-T-2F:Y6	0.75	1.4
PBDB-T-2F:Y6:10%$PC_{71}BM$	1.0	2.0
PBDB-T-2F:Y6:20%$PC_{71}BM$	2.7	3.4
PBDB-T-2F:Y6:30%$PC_{71}BM$	2.4	2.6
PBDB-T-2F:$PC_{71}BM$	7.4	2.7
PBDB-T-2F:IT-4F	2.6	5.6

Supplementary Table 7 | Photovoltaic performance[a] of the PBDB-T-2F:Y6:$PC_{71}BM$ based devices[b] as a function of weight ratios.

PBDB-T-2F:Y6:$PC_{71}BM$	V_{oc} (V)	J_{sc} (mA cm^{-2})	FF (%)	PCE (%)
1:1.2:0	0.82±0.01	25.9±0.2	73.3±0.4	15.7±0.1
1:1.08:0.12 (10%)	0.83±0.01	25.9±0.2	74.6±0.3	16.0±0.2
1:0.96:0.24 (20%)	0.83±0.00	26.0±0.2	75.9±0.6	16.3±0.2
1:0.84:0.36 (30%)	0.84±0.00	25.1±0.2	73.5±0.8	15.5±0.2
1:0:1.2	0.89±0.01	12.9±0.1	59.1±1.5	6.7±0.2

[a]Statistical data obtained from 10 independent devices under AM 1.5 G illumination at 100 mW cm^{-2}. [b]The device architecture is ITO/ZnO/C_{60}-SAM/active layer/MoO_3/Ag.

Supplementary Table 8 | Quantification of voltage losses in the PBDB-T-2F:Y6:20% $PC_{71}BM$ ternary device.

**Device**	E_{opt}^{edge} **(eV)**[a]	$V_{OC, rad}$ **(V)**[b]	$\Delta V_{OC, rad}$ **(V)**[c]	V_{OC} **(V)**	$E_{opt}^{edge}/q - V_{OC}$ **(V)**	$\Delta V_{OC, nr}$ **(V)**[d]
Ternary	1.50 eV	1.08 V	0.42 V	0.83 V	0.67 eV	0.25 V

[a]E_{opt}^{edge} was determined from the intersection of the EQE edge and the local EQE maximum. [b]$V_{OC, rad}$ was calculated from EL and FTPS measurements (Supplementary Fig. 13a) by using the following equation: $V_{OC, rad}=kT/q \ln(J_{SC}/J_{0, rad}+1)$, where $J_{0, rad} = q \int_0^\infty EQE\phi_{bb} dE$. [c] $\Delta V_{OC, rad} = E_{opt}^{edge} - V_{OC, rad}$. [d]$\Delta V_{OC, nr} = V_{OC, rad} - V_{OC}$.

Supplementary References

1 Benduhn, J. *et al.* Intrinsic non-radiative voltage losses in fullerene-based organic solar cells. *Nat. Energy* **2**, 17053, (2017).

2 Nikolis, V. C. *et al.* Reducing Voltage Losses in Cascade Organic Solar Cells while Maintaining High External Quantum Efficiencies. *Adv. Energy Mater.* **7**, 1700855, (2017).

3 Wang, Y. *et al.* Optical Gaps of Organic Solar Cells as a Reference for Comparing Voltage Losses. *Adv. Energy Mater.* **8**, 1801352, (2018).

4 Liu, X. *et al.* Efficient Organic Solar Cells with Extremely High Open-Circuit Voltages and Low Voltage Losses by Suppressing Nonradiative Recombination Losses. *Adv. Energy Mater.* **8**, 1801699, (2018).

5 Baran, D. *et al.* Reduced voltage losses yield 10% efficient fullerene free organic solar cells with >1 V open circuit voltages. *Energy Environ. Sci.* **9**, 3783-3793, (2016).

6 Qian, D. *et al.* Design rules for minimizing voltage losses in high-efficiency organic solar cells. *Nat. Mater.*, (2018).

7 Liu, J. *et al.* Fast charge separation in a non-fullerene organic solar cell with a small driving force. *Nat. Energy* **1**, 16089, (2016).

8 Yuan, J. *et al.* Enabling low voltage losses and high photocurrent in fullerene-free organic photovoltaics. *Nat. Commun.* **10**, 570, (2019).
